# Going along with or taking along with: a cooperation continuum in autism?

**DOI:** 10.3389/fpsyg.2014.01266

**Published:** 2014-11-05

**Authors:** Nicola Yuill

**Affiliations:** ChaTLab, School of Psychology, University of SussexBrighton, UK

**Keywords:** cooperation, development, autism, social interaction, collaboration

We welcome Fantasia et al.'s (FDF's) embodied perspective on cooperation and agree that the definition and varieties of cooperative activities need unpicking. We commend FDF's multidisciplinary approach, offering diverse methods and standards of support, from controlled experimental set-ups and ethological observations to rich descriptions of interaction. Can these approaches work together, or are they incommensurable? We think work in technology-supported collaboration in autism offers new insights.

FDF argue that autistic children are involved in many cooperative exchanges and suggest working from these “positive” perspectives. We propose distinguishing such “cooperative” exchanges from the carefully-constructed cooperative tests used in research with toddlers, by drawing on research which compares different designs of collaborative technology. Such technology is nothing artificial or exotic, just a means of understanding and altering environments to make it easier or harder for organisms to engage in cooperative interactions, e.g., co-working with an interactive surface placed horizontally vs. vertically. Studying people's interactions with different designs tells us about human interactional capacities and processes.

FDF define cooperation broadly, e.g., including echolalic productions and everyday interactions in autism. They claim that such behaviors show “ways of engaging” or signal ongoing engagement. Do we take these behaviors as deliberate signals of engagement, or as merely interpreted by the other as engagement, which might then bootstrap the development of intentional engagement in the child (a strong tradition in developmental psychology: Kaye, 1982)? A useful heuristic is seeing cooperation on a continuum between “going along with” and “taking along with”—committing to considering the interaction, rather than the individual, as unit of analysis.

FDF suggest children with autism are involved in cooperation in everyday social interactions. There just *is* a degree of cooperation between parent and child: it might be difficult to get your child through the regimented routine of a school day, but it would be impossible if the child offered no cooperation. Parents and schools typically work hard to scaffold this basic cooperation, e.g., using visual timetables representing each step, or shaping behavior through reward regimes. But these examples of cooperation, which we term “going along with,” are asymmetrical, with the child often required to comply with the needs of the adult world, rather than having shared goals. An example at the other end of the continuum lies in therapies such as Intensive Interaction, which focus on an adult following and adapting to the child's actions, in the hope of the child recognizing the therapist's behavior as a response contingent on the child's behavior: the child is given the power of eliciting such responses, “taking the adult along with” them. The fact that these interventions produce “engagement” by observer judgment (Escalona et al., 2002) suggests that cooperation (=taking along with) is possible for many autistic children. Synchronization is primarily therapist-driven, but the direction is determined more by the child.

The facet of cooperation that involves engaging the other in joint action seems minimal or absent, both in experimental studies of cooperation in autism and in descriptions of everyday behavior of children with autism. We could interpret this as being a “deficit” of autism, lacking spontaneous intrinsic social motivation, unlike typically-developing children, who quickly adopt ideas about what role the other should play in an interaction, and enforce even relatively novel norms of behavior (Schmidt and Tomasello, 2012). Cooperative novice-expert interactions are typically smooth, but their mechanics can be revealed by observing breaches, e.g., still-face paradigms, participants with autism. This should help in investigating dynamical “taking along with,” given novice-expert pairings work together so invisibly smoothly. Our (Holt and Yuill, 2014) studies of paired children *both* on the autism spectrum enabled us to address both “going along with” and “taking along with,” given that the children have to collaborate with each other, rather than with a compliant and strongly-scaffolding other. We demonstrated contingent action between such pairings using a dual-control game that stalled progress until participants' responses matched. Active other-awareness occurred here, but not in a similar setting without constraints to support contingency. Thus, the children showed collaborative capacity only in environments constrained to support it. Subsequent work-in-progress with touch-technology further clarifies three prerequisites for any collaboration to occur: understanding the activity (i.e., criteria for performing tasks), coordinating action with the partner (going along with), and fostering coordination of the other's activity with their own (taking along with). For pair success, both children must understand the goal of the activity itself and at least one child must be able to coordinate his behavior with the partner's, even if the partner cannot reciprocate. Thus, children could successfully play together if just one child could follow or match his behavior to that of a less-able child not displaying any contingent behavior. However, a more complex form of collaboration is required if there is a further constraint, of shared solutions being correct. With such a constraint, then at least one partner needs to realize this and to bring his partner along to the right solution, necessitating “mutual engagement… in a coordinated effort” (Roschelle and Teasley, 1995, p. 70).

We argue that “going along with”/“taking along with” marks a useful *gradation* in cooperation, encompassing both FDF's “everyday cooperative interaction” and the more structured requirements of lab-based cooperative tasks. A sharp dichotomy between the broader idea of engagement as a prerequisite of cooperation and the narrower focus on agreed, planful, outcome-directed joint working, loses the benefit of the enactive approach in uniting literature across paradigms and blurring the classical motivation–cognition divide. We must reconcile top-down theoretical claims about prerequisites of “true” collaboration with questions driven by observation of everyday behavior, to consider similarities and differences in cooperative encounters in different groups of participants (e.g., toddlers, people with autism) and to characterize the place, in collaborative activity, of a sense of joint engagement, from second- and third-person perspectives. The debate underlines the need, in studying cooperation, to consider the behavior of both participants in relation to each other; it is the interaction that is cooperative, not only the participants.

## Conflict of interest statement

The author declares that the research was conducted in the absence of any commercial or financial relationships that could be construed as a potential conflict of interest.
